# Human papillomavirus type 18 E5 oncoprotein cooperates with E6 and E7 in promoting cell viability and invasion and in modulating the cellular redox state

**DOI:** 10.1590/0074-02760190405

**Published:** 2020-03-16

**Authors:** Jimena Hochmann, Felipe Parietti, Jennyfer Martínez, Ana C Lopez, Mara Carreño, Celia Quijano, Enrique Boccardo, Laura Sichero, Matías N Möller, Santiago Mirazo, Juan Arbiza

**Affiliations:** 1 Universidad de la República, Facultad de Ciencias, Sección Virología, Montevideo, Uruguay; 2 Universidad de la República, Facultad de Medicina, Centro de Investigaciones Biomédicas, Departamento de Bioquímica, Montevideo, Uruguay; 3 Universidad de la República, Facultad de Ciencias, Instituto de Química Biológica, Laboratorio de Fisicoquímica Biológica, Montevideo, Uruguay; 4 Universidade de São Paulo, Instituto de Ciências Biomédicas, Departamento de Microbiologia, São Paulo, SP, Brasil; 5 Hospital das Clinicas da Faculdade de Medicina da Universidade de São Paulo, Centro de Investigação Translacional em Oncologia, Instituto do Câncer do Estado de São Paulo, São Paulo, SP, Brasil

**Keywords:** HPV-18 E5/E6/E7, cooperation, cell invasion, redox state, cellular transformation, reactive oxygen species.

## Abstract

**BACKGROUND:**

High-risk human papillomaviruses (HR-HPVs) are the etiological agents of cervical cancer. Among them, types 16 and 18 are the most prevalent worldwide. The HPV genome encodes three oncoproteins (E5, E6, and E7) that possess a high transformation potential in culture cells when transduced simultaneously. In the present study, we analysed how these oncoproteins cooperate to boost key cancer cell features such as uncontrolled cell proliferation, invasion potential, and cellular redox state imbalance. Oxidative stress is known to contribute to the carcinogenic process, as reactive oxygen species (ROS) constitute a potentially harmful by-product of many cellular reactions, and an efficient clearance mechanism is therefore required. Cells infected with HR-HPVs can adapt to oxidative stress conditions by upregulating the formation of endogenous antioxidants such as catalase, glutathione (GSH), and peroxiredoxin (PRX).

**OBJECTIVES:**

The primary aim of this work was to study how these oncoproteins cooperate to promote the development of certain cancer cell features such as uncontrolled cell proliferation, invasion potential, and oxidative stress that are known to aid in the carcinogenic process.

**METHODS:**

To perform this study, we generated three different HaCaT cell lines using retroviral transduction that stably expressed combinations of HPV-18 oncogenes that included HaCaT E5-18, HaCaT E6/E7-18, and HaCaT E5/E6/E7-18.

**FINDINGS:**

Our results revealed a statistically significant increment in cell viability as measured by MTT assay, cell proliferation, and invasion assays in the cell line containing the three viral oncogenes. Additionally, we observed that cells expressing HPV-18 E5/E6/E7 exhibited a decrease in catalase activity and a significant augmentation of GSH and PRX1 levels relative to those of E5, E6/E7, and HaCaT cells.

**MAIN CONCLUSIONS:**

This study demonstrates for the first time that HPV-18 E5, E6, and E7 oncoproteins can cooperate to enhance malignant transformation.

Human papillomaviruses (HPVs) are a heterogeneous group of small, non-enveloped, circular, double-stranded DNA viruses that infect the epithelia of the skin and mucosa.^1^ ^,^ ^2^ To date, over 200 types have been identified and characterised.^3^ Most viral types from the α genus infect mucosal tissues and, among these, the high-risk (HR) HPVs 16 and 18 account for approximately 70-80% of cervical cancer cases worldwide. Globally, the most prevalent HPV types found in premalignant lesion and in cervical cancer are HPV-16 (46-63%) and HPV-18 (10-14%).^4^ ^,^ ^5^ Furthermore, HPV-18 accounts for approximately 12% of squamous cell carcinoma (SCC) and 37% of cervical adenocarcinoma (ADC).^6^

In this context, HR-HPV oncoproteins E5, E6, and E7 are the primary viral factors responsible for the initiation and progression of cervical cancer. The extensively studied HR-HPV E6 and E7 oncoproteins are constitutively expressed in cancer cells following viral DNA integration and target key cellular tumour suppressor proteins such as p53 and pRb, respectively, where they abrogate their activities and thus, contribute to unchecked cell cycle progression, genomic instability, and cell immortalisation.^7^ ^,^ ^8^ ^,^ ^9^ ^,^ ^10^ ^,^ ^11^ Several studies have demonstrated the transformation potential of these oncoproteins in cell lines of diverse origins,^8^ ^,^ ^12^ ^,^ ^13^ and this transformation is primarily associated with the disruption of a variety of signal transduction pathways that are crucial for cell homeostasis.^14^ ^,^ ^15^ ^,^ ^16^ ^,^ ^17^ Of particular interest is the MAPK/ERK pathway that governs the signal transduction of mitogenic growth factor receptors such as epithelial growth factor receptor (EGFR). It has been reported that HR-HPV E5 oncoproteins enhance ligand-dependent activation of EGFR that elicits signals that are transduced via the MAPK/ERK pathway and to ultimately lead to the expression of genes related to cell proliferation.^18^ ^,^ ^19^ ^,^ ^20^ Although the E5 ORF is deleted in advanced cervical lesions, it is believed that the encoded protein actively contributes during the early phases of carcinogenesis, making E5 along with E6 and E7 particular targets of interest in cervical cancer prevention and treatment.^21^ HR-HPV E5 has been shown to cooperate positively with E6 and E7 in cancer progression to enhance their transformation activity in the early stages of precancerous lesions.^21^ It has also been observed that E5 exhibits cell transformation potential when expressed alone; however, this transformation potential is weak.^20^ In this study, we focused on examining the effects of HPV-18 oncoproteins on certain hallmarks of cancer in HaCaT cells. HPV-18 is the second most prevalent HPV type in cervical cancer worldwide; however, the genotype-specific oncogenic mechanisms remain largely unknown.

Other factors that may contribute to cervical carcinogenesis include chronic inflammation and oxidative stress. These processes may induce the production of reactive oxygen species (ROS) that exert harmful effects on cells and create favourable conditions for malignant transformation.^22^ It has been observed that the E5, E6, and E7 viral oncoproteins are involved in inducing chronic inflammation associated with cervical cancer. For example, it has been reported that the presence of these proteins may lead to increased cyclooxygenase-2 (COX-2) expression.^23^ Additionally, cells infected with HR-HPVs possess the ability to adapt to oxidative conditions by increasing the levels of protective antioxidants such as glutathione and enzymes such as catalase and peroxiredoxins, ultimately favouring cancer cell survival.^24^ However, the mechanisms by which these pleiotropic oncoproteins cooperate and enhance their oncogenic potential are still under investigation.^25^ ^,^ ^26^ ^,^ ^27^

To date, the majority of the studies addressing HR-HPV E5, E6, and E7 oncoproteins properties have focused on those encoded by HPV-16. HPV-18 is the second most prevalent HPV type globally.^4^ ^,^ ^28^ In the present study, we aimed to evaluate the effect of HPV-18 oncoproteins on key aspects of malignant transformation by developing a spontaneously immortalised human keratinocytes (HaCaT) cell-based system that stably expresses the E5, E6, and E7 oncogenes from this HPV type. We focused on the examination of certain features of malignant transformation such as cell proliferation, viability, and cell invasion potential process, and we assessed the ability of these proteins to modulate the cell redox state.

## MATERIALS AND METHODS

*Cell lines* - Spontaneously immortalised human keratinocyte (HaCaT) cells were purchased from Banco de células do Rio de Janeiro (BCRJ), Brazil (batch number 001071, certificate of analysis provided by the supplier) and maintained in dulbecco’s modified eagle’s medium (DMEM) low glucose medium (Capricorn, Ebsdorfergrund, Germany) supplemented with 10% foetal bovine serum (FBS) (Gibco, Massachusetts, USA). Bosc23 ecotropic and Am-12 amphotropic cells were maintained in DMEM supplemented with 10% FBS and antibiotics.

*Plasmids and Retroviral transductions* - HaCaT cells used in this study were tested internally for mycoplasm by polymerase chain reaction (PCR). HaCaT E5/E6/E7 cells were obtained through co-infection with a retroviral vector carrying the MSCV-N-puro-18E5 plasmid (Addgene # 37882, Massachusetts, USA) and with a pLXSN retroviral vector that contained cloned HPV-18 E6/E7genes and was kindly provided by Dra, Sichero from Instituto do Câncer do Estado de São Paulo. Briefly, 15 µg of each plasmid were used to transfect the packaging ecotropic Bosc23 cells using the FuGENE® 6 Transfection Reagent (Promega, Wisconsin, USA). Transfection of Bosc23 was performed to produce a transient virus stock. After 48 h, cell supernatants in the presence of 10 mg mL-1 of polybrene (TR-1003, Sigma Aldrich, Missouri, USA) were used to transduce the amphotropic packaging cell line Am 12 to obtain supernatants possessing high retroviral particle titres. At 48 h post infection, Am12 cells that were transduced with pLXSN HPV18-E6/E7 were selected using 0.5 mg mL-1 G418 (Gibco, Massachusetts, USA) for one week until the death of the control cells (non-transduced Am12 cells treated with G418). Am12 cells that were transduced with the MSCV-N-puro-18E5 retroviral vector were selected using 0.5 µg mL-1 of Puromycin (Santa Cruz Biotechnology, Texas, USA) for one week until the death of the control cells (non-transduce Am12 cells treated with puromycin).

Viral stocks were titrated according to a NIH3T3 cells G418-resistant colony assay.^29^ A heterologous retroviral promoter was used to drive both E6 and E7 expression to facilitate the normalisation of protein levels among infected HaCaT cells. For E5, a PGK-1 promoter that can efficiently drive high levels of expression of the target protein was used.

Equal amounts of all retrovirus preparations were used to infect HaCaT cells (at MOI = 10) in the presence of 10 mg mL-1 of polybrene. HaCaT cells were infected with retroviral particles containing the vector harbouring pLXSN E6/E7 HPV-18, and they were selected using 0.5 mg mL-1 G418 (Gibco, Massachusetts, USA) for one week or until the non-transduced control cells died. Cells infected with the retroviral vector containing E5 HPV-18 were selected using 0.5 µg mL-1 of Puromycin (Santa Cruz Biotechnology, Texas, USA) for one week or until the non-transduced control cells died. To obtain HaCaT E5/E6/E7 cells, a co-transduction was performed using cell supernatants from Am 12 cells transduced with MSCV-N-puro-18E5 vector and cell supernatants from Am 12 transduced with pLXSN E6/E7 HPV-18. Co-transduced HaCaT cells were initially selected in 0.5 mg mL-1 G418 for one week and then with 0.5 µg mL-1 of puromycin for an additional week.

*RNA extraction and reverse transcription-PCR (RT-PCR)* - Total cellular RNA was extracted using TRIzol® (Sigma Aldrich, Missouri, USA). First-strand complementary cDNAs were generated by reverse transcription from 2 µg of total RNA in a total volume of 20 µL using the High Capacity RNA cDNA kit (Life Technologies, California, USA). One uL of cDNA (100 ng µL-1) was amplified in a 25 µL total volume PCR reaction containing 1X PCR buffer, 1.2mM MgCl2, 0.16 mM dNTPs, 0.2µM of each primer, and 1U of Ampli Taq Gold (AppliedBiosystems, California, USA). cDNA samples from cells transduced with HPV-18E5, HPV-18E6/E7, and HPV-18 E5/E6/E7 were amplified in a 25 µl total volume PCR reaction using primers specific for E7-HPV-18 under the following cycling conditions: 95ºC for 5 min, 94ºC for 30 s, and 35 cycles at 53ºC for 30 s, 72ºC for 30 s, and 72ºC for 7 min. E7-HPV18F: 5´ATGTCACGAGCAATTAAGC3´, E7-HPV18R: 5´ TTCTGGCTTCACACTTACAACA3´. For the amplification of HPV-18 E5, a PCR reaction was performed using specific primers under the following cycling conditions: 95ºC for 5 min, 94ºC for 30 s, and 35 cycles at 60ºC for 30 s, 72ºC for 30 s, and 72ºC for 7 min. E5-HPV18F: 5’ CATGTATGTGTGCTGCCATG3´, E5-HPV18R: 5´GGCAGGGGACGTTATTACCA 3´, GADPHF: 5´TGCACCACCAACTGCTTAGC3´, GADPHR: 5´GGCATGGACTGTGGTCATGAG3´. GAPDH cDNA amplification was performed to assess cDNA quality under the following cycling conditions: 95ºC for 5 min, 94ºC for 30 s, and 35 cycles at 60ºC for 30 s, 72ºC for 30 s, and 72ºC for 7 min. HF (Human Foreskin Keratinocytes)^30^ cells expressing the complete genome of HPV-18 were used as a positive control for the RT-PCR assays. The PCR reaction concentrations were identical to those described above. All reactions were performed in a Veriti™ 96-Well Thermal Cycler PCR machine (Applied Biosystems/Thermo Fisher Scientific, Massachusetts, USA), and amplified products were analysed using 3% agarose gel electrophoresis and were stained with ethidium bromide.

*Viability assay* - Cell viability was assessed using 3-(4, 5-dimethylthiazol-2-yl)-2, 5-diphenyl tetrazolium bromide) (MTT). Briefly, 8 × 10^3^ cells from each of the four cell lines (HaCaT, HaCaT E5, HaCaT E6/E7, and HaCaT E5/E6/E7) were seeded at day 0 into 96-well plates at 37ºC under 5% CO_2_. After 48 h (Day 2), the cells were stained with a 5 mg mL^-1^ solution of MTT (Sigma-Aldrich, Missouri, USA). After 4 h, 100 μL of dimethylsulfoxide (DMSO) was added to each well to dissolve the formazan crystals. Finally, the absorbance was measured at 570 nm using a Varioskan FLASH (Thermo Fischer Scientific, Massachusetts, USA).

*Proliferation assay* - A cell proliferation curve was performed by seeding 5.0 × 10^4^ cells (day 0) from each stable HaCaT cell line (HaCaT E5-18; HaCaT E6/E7-18, and HaCaT E5/E6/E7-18) into 6-well plates. Viable cells were counted in a Neubauer chamber after staining with 0.4% Trypan Blue and the number of cells corresponding to each day of culture (four days) was determined. Each cell line was plated in triplicate in two independent experiments.

*Invasion assay* - The invasion potential of HaCaT, HaCaT E5, HaCaT E6/E7, and HaCaT E5/E6/E7 was determined using the QCM™ Collagen Cell Invasion Assay (Millipore, Darmstadt, Germany) following the manufacturer’s instructions. Briefly, 9 × 10^5^ HaCaT cells were seeded into the upper chamber of a transwell permeable support membrane insert that was pre-wetted with DMEM containing 1% of FBS. The bottom chamber was supplemented with DMEM containing 10% FBS, and the plates were incubated for 48 h at 37ºC. Cells were stained using the solution provided by the supplier, and invasion was initially assessed microscopically using a Leitz Labovert FS inverted microscope (Oberkochen, Germany). Next, the staining solution was removed, and the absorbance at 560 nm was measured using a Varioskan FLASH (Thermo Fischer Scientific, Massachusetts, USA).

*ROS quantification* - Intracellular oxidant levels were measured within the four cell lines (HaCaT, HaCaT E5, HaCaT E6/E7, and HaCaT E5/E6/E7) using the fluorescent probe 5-(and-6)-chloromethyl-2’-7’-dichlorodihydrofluorescein diacetate (CM-H_2_DCFDA) (Thermo Fischer Scientific, Massachusetts, USA). This sensitive but non-specific probe for ROS is a chloromethyl derivative of 2’-7’-dihydrofluorescein diacetate (H_2_DCFDA).^31^ CM-H_2_DCFDA passively diffuses into cells, where its acetate groups are cleaved by intracellular esterases and the thiol-reactive chloromethyl moiety reacts with intracellular thiols. Subsequent oxidation yields the fluorescent adduct 2’-7’-dichlorofluorescein (DCF) that is retained inside the cell, thus facilitating long-term studies.^31^ CM-H2DCFDA (10 μM) dissolved in DMEM without phenol red (Sigma-Aldrich, Missouri, USA) was added to a 6-well plate containing 1 × 10^4^ cells per well. Plates were incubated for 30 min at 37ºC, and cells were analysed by flow cytometry using a BD FACS Calibur cytometer.

*Catalase activity assay* - Total protein extracts were obtained using RIPA lysis buffer (20 mM Tris-HCl, pH 7.5, 150 mM NaCl, 0.5% sodium deoxycholate, 1% NP-40, and 0.1% SDS) supplemented with complete protease inhibitor cocktail (Roche, Basel, Switzerland). Samples from the four cell lines were centrifuged, and the supernatants were used to measure enzyme activity. Catalase activity was assayed spectrophotometrically according to the decomposition of 10 mM H_2_O_2_ by catalase that was present in the samples at 240 nm (ε _(240 nm)_ = 39.4 M^-1^cm^-1^).^32^ To achieve this, dilutions of cell lysates were used that possessed concentrations ranging from 0.75 µg µL^-1^ to 1.0 µg µL^-1^ of total protein in Hank’s Balanced Salt Solution (HBSS). Absorbance measurements were conducted at room temperature in a UV-Vis Varian Cary 50 spectrophotometer for 2 min or until the absorbance value decreased by 10% compared to the initial rate conditions. Catalase enzymatic activity was expressed as U.mg^-1^ of protein.

*Glutathione (GSH) quantification* - Total glutathione within cell lysates was quantified by HPLC in a manner similar to that described by Amen et al.^33^ Briefly, 20 µL of lysate was added to 10 µL of 50 mM ammonium bicarbonate (pH 7.8) and 10 µL of 4 mM triscarboxyethylphosphine (TCEP), and this solution was mixed and incubated for 10 min at 22-24ºC. Next, 10 µL of 20 mM monobromobimane dissolved in acetonitrile was added to the solution, and the solution was then mixed and incubated for 10 min at room temperature. Then, the samples were diluted by addition of 100 µL of 0.1% trifluoroacetic acid dissolved in water and filtered through 5 kDa MWCO filters (GE Healthcare, Illinois, USA). The filtrate was then collected, and 5 µL was injected into an HPLC by manual injection. The HPLC consisted of a quaternary pump (Agilent 1260 VL, California, USA) and a diode array detector (Agilent 1260 DAD VL, California, USA). An Ascentis C18 (10 cm × 4.6 mm, 3 µm) column was used (Supelco, Sigma-Aldrich, USA). The method of separation incorporated the initial use of 0.1% TFA in water followed by gradually increasing the proportion of acetonitrile. A calibration curve was constructed using standard glutathione derivatised with monobromobimane, and cell samples were quantified by the area under the curve based on the absorbance at 396 nm and the corresponding dilutions.

*Western blot* - HaCaT cell pellets obtained from the four cell lines (HaCaT, HaCaT E5, HaCaT E6/E7, and HaCaT E5/E6/E7) were washed in ice cold phosphate buffered saline (PBS) and centrifuged, and protein lysates were then extracted by incubating on ice for 30 min in RIPA buffer (20 mM Tris-HCl, pH 7.5, 150 mM NaCl, 0.5% sodium deoxycholate, 1% NP-40, 0.1% SDS) containing complete protease inhibitor cocktail (Roche, Basel, Switzerland). For protein analysis, 50 µg of the protein extracts were loaded into 12 % SDS polyacrylamide gels, electrophoresed, and transferred to PVDF membranes (GE Healthcare, Buckinghamshire, UK). Membranes were blocked with TBS (1X), containing 5% BSA and 0.6% Tween 20 for 1 h, and they were then incubated with primary antibody specific for peroxiredoxin 1 (Anti-PRX1) that was diluted 1/1000 in TBS (1X) containing 5% BSA and 0.1% Tween 20 (AB Frontier, LF PA0086, Seoul, Korea). Immunocomplexes were detected by subsequent incubation with anti-rabbit polyclonal Alexa 680-conjugated secondary IgG antibodies using G: BOX equipment (SYNGENE, Cambridge, UK). The PRX1 signal was normalised to total protein loading to avoid possible effects of E5/E6/E7 expression on housekeeping gene expression.^34^

*Proteome profiling of keratinocytes transduced with HPV18 oncogenes* - The relative intensities of 84 different proteins involved in different signalling-mediated pathways related to cancer were evaluated using the Human XL Oncology Array Kit as suggested by the manufacturer (R&D Systems, MN, USA, ARY026). Protein levels were quantified using the ImageQuant TL software (GE Healthcare, Buckinghamshire, UK), and the individual Western blot membranes were normalised according to the pixel densities of the 6 reference spots. Each experiment was performed in duplicate.

*Statistical analysis* - Statistical analyses and graphical presentations were conducted using GraphPad Prism version 8.0.1 (244) software (GraphPad Software Inc., San Diego, CA, USA). All experiments were performed in triplicate, and data were presented as the mean ± standard deviation (SD). Data were analysed by One-Way unpaired ANOVA followed by Tukey’s HSD *post-hoc* test, with the exception of data retrieved from the Human XL Oncology protein array, which was analysed using a two-way ANOVA test to assess statistical significance among groups along with Dunnett *post-hoc* test for intra group comparison. Statistical significance was determined at *p* < 0.05.

## RESULTS

**Fig. 1:**
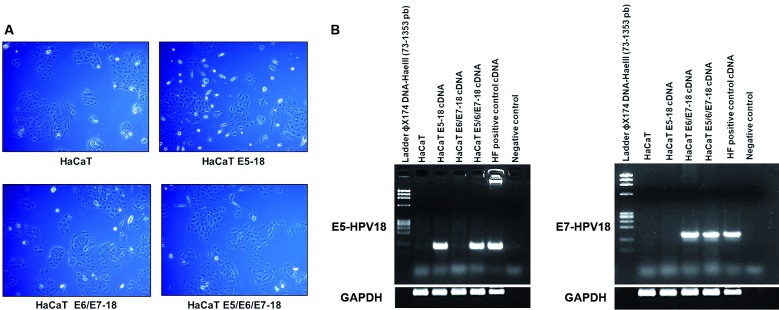
characterisation of stable HaCaT cell lines transduced with viral HPV-18 E5, E6, and E7. (A) Morphologies of the four cell lines (HaCaT, HaCaT E5, HaCaT E6/E7, and HaCaT E5/E6/E7) were analysed using a Zeiss Primovert microscope (Zeiss, Jena, Germany). Magnification 10x. (B) Left: reverse transcription-polymerase chain (RT-PCR) for E5 oncogene in four HaCaT cells lines. Right: RT-PCR for E7 in four HaCaT cells lines. Human Foreskin Keratinocytes expressing the HPV-18 whole genome (HF) DNA were employed as positive control for RT-PCR assays.

*Transduction of HaCaT cells with HPV-18 E5, E6, and E7* - HaCaT cells transduced with the viral HPV-18 oncogenes did not exhibit major differences in morphology compared to that of control HaCaT cells; however, we observed that some cells transduced with E5 presented an elongated and flattened morphology compared to that of cells transduced only with E6/E7. Additionally, cells that were co-transduced with E5/E6/E7 exhibited an intermediate phenotype between that of E6/E7 and cells transduced with E5 only (Fig. 1A). To confirm the expression of E5 and E6/E7 oncogenes in co-infected cells, we performed RT-PCR using specific primers that amplify fragments of the E5 and E7 HPV-18 genes. Amplicons of the expected size (103 and 137 bp, respectively) were observed (Fig. 1B).

**Fig. 2:**
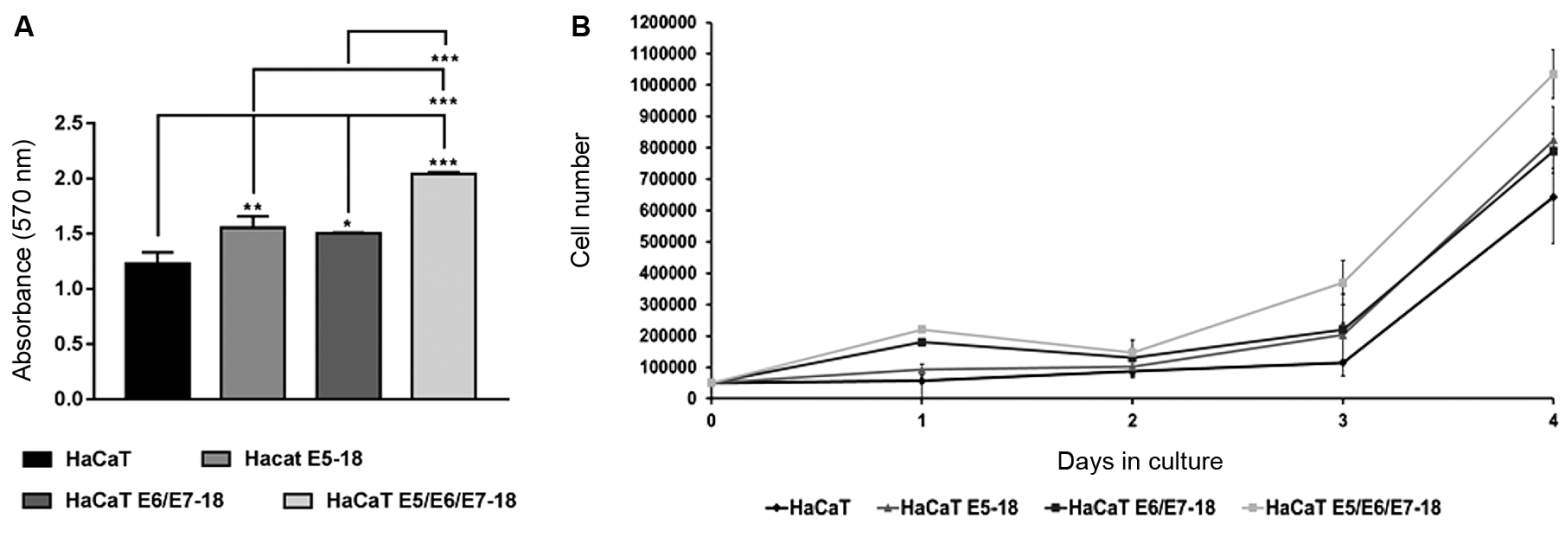
effect of E5, E6, and E7 oncogenes on HaCaT cell viability and cell proliferation. (A) A total of 8.0 × 10^3^ HaCaT parental cells or human papillomavirus (HPV)-18 transduced cells were seeded into 96-well plates for 48 h at 37ºC under 5% CO_2_, and the absorbance at 570 nm was measured spectrophotometrically. Three independent experiments were performed in triplicate. Mean ± standard deviation (SD) is shown for each cell line. A one-way analysis of variance (ANOVA) test was conducted to assess statistical significance among groups along with Tukey’s HSD test for intra group comparison. (*) p < 0.05, (**) p < 0.01, (***) p < 0.001. (B) Cell proliferation was evaluated by counting viable cells within the Neubauer Chamber. We plated 5.0 × 10^4^ cells (day 0) from the stable HaCaT cell lines (HaCaT E5-18; HaCaT E6/E7-18, and HaCaT E5/E6/E7-18) into 6-well plates, and each plate was numbered corresponding to a different day of the experiment (day 1, day 2, day 3, and day 4). Plates were incubated at 37ºC under 5% CO_2_ overnight. The cells were counted in the Neubauer chamber corresponding to each day. After four days of culture, a growth curve was generated using Microsoft Excel. Each cell line was plated in triplicate for three independent experiments.

**Fig. 3:**
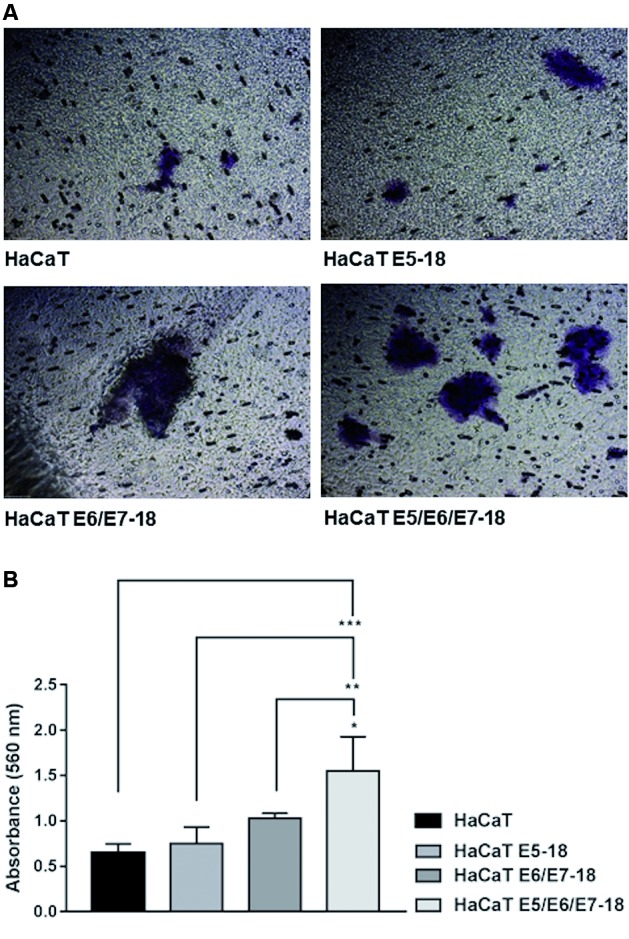
effect of human papillomavirus (HPV)-18 E5, E6, and E7 oncogenes on invasion in HaCaT cells. Invasive potential was assessed using a QCM™ Collagen Cell Invasion Assay. (A) A total of 9.0 × 10^5^ starved cells were seeded into each chamber containing serum-free dulbecco’s modified eagle’s medium (DMEM) low glucose medium. The bottom chamber was filled with DMEM low glucose 10% foetal bovine serum (FBS) that acted as a chemoattractant, and the plates were incubated at 37°C under 5% CO_2_ for 48 h. Invading cells were visualised under a Leitz Labovert FS inverted microscope (Oberkochen, Germany) at a magnification of 10x. Two independent experiments were performed in triplicate, and the magnification was 10x (B). Quantification of results obtained in (A) measured by absorbance at 560 nm. The mean ± standard deviation (SD) is shown. A one-way analysis of variance (ANOVA) test was conducted to assess statistical significance among groups along with Tukey’s HSD test for intra-group comparison. (*) p < 0.05, (**) p < 0.01, (***) p < 0.001.

*Effect of the viral oncogenes on cell viability, proliferation, and invasion* - The effect of HPV-18 oncogenes on the viability of HaCaT cells was determined using an MTT assay. Our results revealed that HaCaT cells transduced with HPV-18 E5 or with HPV-18 E6/E7 or cells co-transduced with all three viral oncogenes exhibited a significantly higher absorbance at 570 nm than did control HaCaT cells (Fig. 2A). This effect was more evident in cells expressing the three HPV-18 oncogenes (Fig. 2A). No differences in MTT reduction activity were observed between HaCaT cells expressing HPV-18 E5 and HPV-18 E6/E7. These results suggest the existence of an increase in the number of viable cells, particularly in cells expressing E5/E6/E7, compared to that of control cells. As changes in cell metabolism cannot be discarded as potential sources of differences in MTT reduction, we further analysed cell proliferation potential by performing growth curves. As shown in Fig. 2B, we observed that HaCaT E5/E6/E7-18 cells proliferated faster than the parental HaCaT cells, HaCaT E5, and HaCaT E6/E7 cells during the four days in culture. These observations corroborate and complement the results obtained in the MTT assay. Another important feature involved in the process of carcinogenesis is the invasion of local tissues through the degradation of the extracellular matrix (ECM). The acquisition of invasive potential is an important step in local tumour spread and metastasis to distant sites. To evaluate the effect of HPV-18 oncogenes on the invasion capacity of HaCaT cells, we performed a transwell invasion assay using a collagen matrix. As shown in Fig. 3A, HaCaT cells transduced with E5/E6/E7 exhibited a higher number of invading cells (stained cells) through the collagen matrix compared to the invasion observed from HaCaT, HaCaT E5, and HaCaT E6/E7 cells, and these cells also exhibited higher levels of cell invasion as measured by absorbance at 560 nm (Fig. 3B). These results indicate that the three HPV-18 oncogenes cooperate significantly to induce an increase in the invasion capacity of HaCaT cells.

**Fig. 4:**
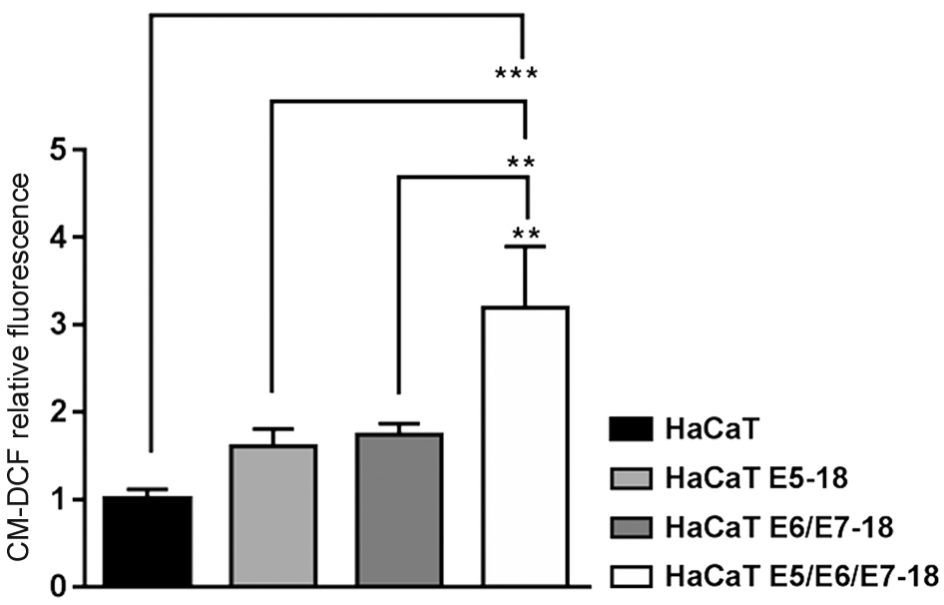
modulation of E5, E6, and E7 oncogenes from human papillomavirus (HPV)-18 in the production of reactive oxygen species (ROS) in HaCaT cells. ROS were assessed by measuring CM-DCF fluorescence using flow cytometry in a BD FACSCalibur cytometer. Geometrical mean fluorescence intensity of the cell population was obtained and expressed relative to control values (n = 3 culture dishes per group). Results are the mean ± standard deviation (SD). A one-way analysis of variance (ANOVA) test was conducted to assess statistical significance among groups along with Tukey’s HSD test for intra group comparison. (*) p < 0.05, (**) p < 0.01, (***) p < 0.001.

**Fig. 5:**
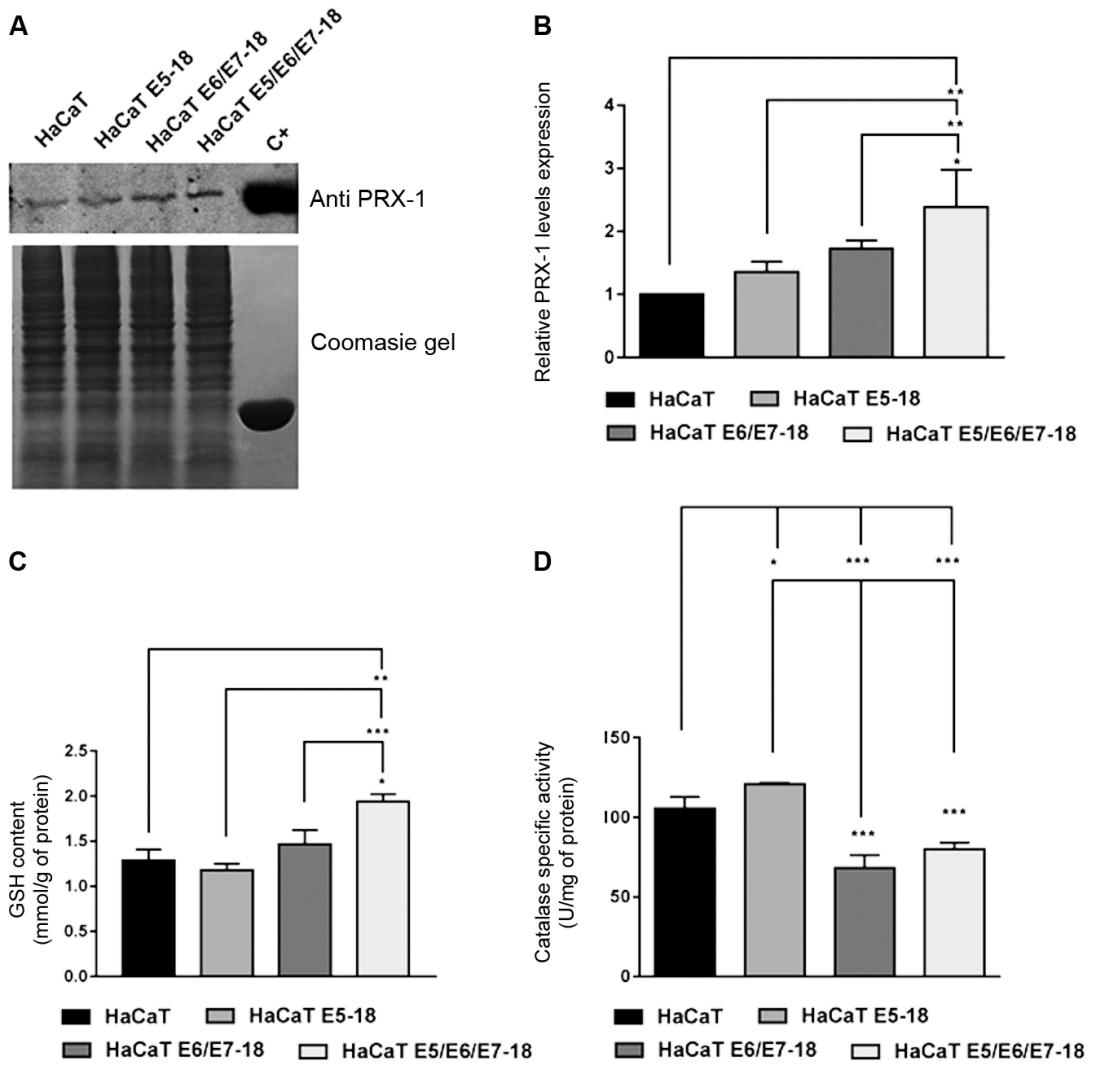
evaluation of the effect of human papillomavirus (HPV)-18 E5, E6, and E7 oncogenes on the expression levels of PRX-1 and on the enzymatic activity of catalase and glutathione (GSH). (A) Representative immunoblot of peroxiredoxin 1 (PRX1) within the four cell lines analysed (HaCaT, HaCaT E5-18, HaCaT E6/E7, HaCaT E5/E6/E7). As a positive control (C+), we used the commercial recombinant protein PRX1. One representative experiment of three independent assays is shown. (B) Densitometric analysis of PRX1 levels in the four cell lines as described above. Values were normalised to Coomasie Gel, and averages ± standard deviations (SDs) of three independent experiments are shown. (C) GSH levels were studied in four HaCaT cells lines. Two independent experiments were performed in triplicate. Mean ± SDs of two independent experiments is shown. (D) Catalase enzymatic activity in parental and HPV-18 transduced HaCaT cells. Two independent experiments were conducted in triplicate. Mean ± SD is shown for each cell line. For all experiments, a one-way analysis of variance (ANOVA) test was conducted to assess statistical significance among groups along with Tukey’s HSD test for intra-group comparison. (*) p < 0.05, (**) p < 0.01, (***) p < 0.001.

*Effect of HPV-18 E5/E6/E7 oncogenes in ROS production, and in the modulation of the antioxidant stress system* - To determine the influence of HPV-18 viral oncogenes on the cellular redox state, ROS production was measured using the CM-H2DCF fluorescent probe. As observed in Fig. 4, HaCaT cells containing HPV-18 E5/E6/E7 viral oncogenes exhibited significantly higher levels of oxidised CM-H2DCF probe (CM-DCF) compared to that observed in HaCaT control cells, HaCaT E5 cells, and HaCaT E6/E7 cells, indicating an increase in intracellular ROS. As it has been previously observed that the E6 oncoprotein of HPV-18 enhances ROS production and decreases catalase activity,^35^ we questioned if this increment in intracellular ROS in HaCaT cells co-transduced with the three oncogenes could be due to a reduction in the function of the cell antioxidant systems. To assess this, we analysed GSH and catalase levels and their enzymatic activity, and we determined the protein levels of peroxiredoxin 1 (PRX1). The combined expression of HPV-18 E5/E6/E7 oncogenes in HaCaT cells was associated with the upregulation of PRX1 expression levels compared to those of HaCaT and HaCaT E5 cells (Fig. 5A, B). HaCaT cells co-transduced with three viral oncogenes also exhibited significantly increased levels of the intracellular low-molecular weight antioxidant GSH compared to the levels observed in all other cell lines (Fig. 5C). By contrast, HaCaT cells transduced with E6/E7 or co-transduced with the three viral oncogenes of HPV-18 (E5/E6/E7) exhibited significantly lower catalase activity compared to that of the HaCaT parental cells or cells solely expressing E5 (Fig. 5D).

**Fig. 6:**
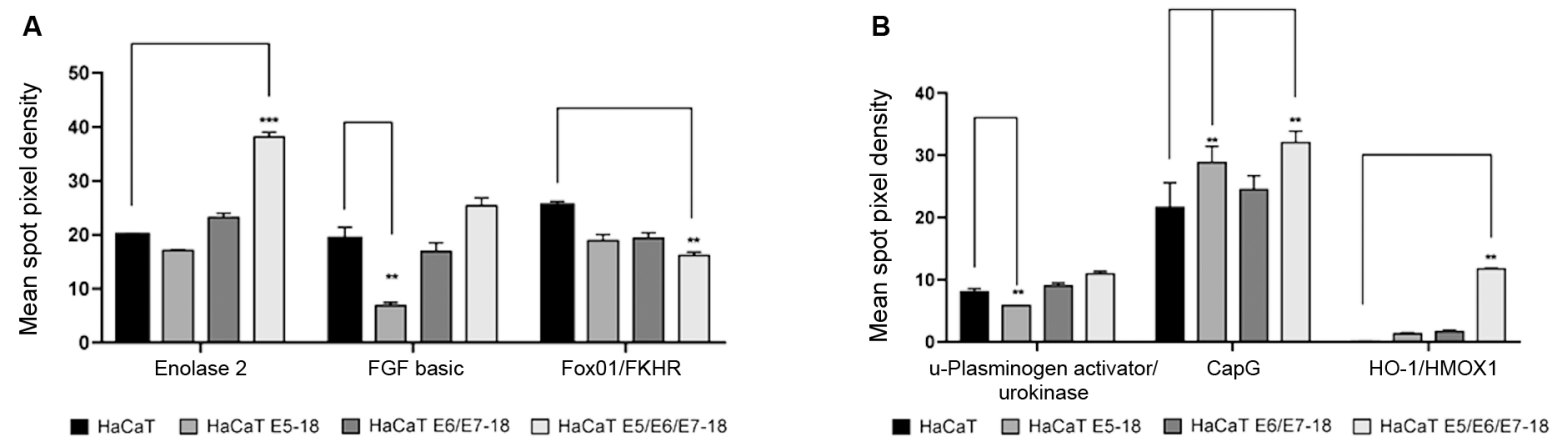
expression profile of proteins involved in certain aspects of cancer development such as cell proliferation, glycolytic metabolism, and cell invasion. (A) Total protein extracts from control and transduced HaCaT cells were obtained using a specific lysis buffer (Lysis buffer 17) (R&D Systems, Catalogue # 895943) included in the array kit. The relative expression levels of three proteins involved in cell proliferation and metabolism pathways (FGF basic, Enolase 2, and Fox01/FKHR) in HaCaT transduced and co-transduced cells compared to that in control cells is shown. (B) Relative expression levels of proteins expressed as mean spot pixel density that are involved in cell invasion (uPA, and CapG) and one protein involved in oxidative stress responses (HO-1/HMOX1) are shown for HaCaT transduced and co-transduced cells compared to those of control cells as assessed by the Human XL Oncology Array Kit (R&D Systems, MN, USA, ARY026). Averages ± standard deviations (SDs) of two independent experiments are shown. A two-way analysis of variance (ANOVA) test was conducted to assess statistical significance among groups along with Dunnett *post-hoc* test for intra-group comparison. (*) p < 0.05, (**) p < 0.01, (***) p < 0.001.

*Cooperation among the HPV-18 E5, E6, and E7 viral oncogenes in the expression profiles of certain proteins involved in cancer development* - The abnormal activation of certain signalling cascades plays a critical role in the development and progression of cancer. Thus, we evaluated the expression levels of 84 proteins that are involved in different pathways related to cancer in spontaneously immortalised keratinocytes that were transduced with viral oncogenes of HPV-18 or that were not transduced. Six proteins were differentially expressed in the four cell lines studied. As shown in Fig. 6A, we observed statistically significant elevated expression of Enolase2, a protein involved in glycolytic metabolism, in cells expressing the HPV-18 E5, E6, and E7 oncogenes compared to expression levels in the HaCaT control. In these cells, we also observed a slight increase in the expression levels of the transcription factor FGF basic compared to that of the control and a significant reduction in the expression of FGF basic in HaCaT E5 cells compared to that in the HaCaT controls. In contrast, the expression of the transcription factor Forkhead box protein O1 (Fox01/FKHR) was significantly reduced in HaCaT E5/E6/E7 cells compared to that in the control HaCaT cells (Fig. 6A). Additionally, in the same protein array, we observed significantly higher expression levels of Capping actin protein (CapG), a protein involved in angiogenesis and cell invasion, in cells expressing the three HPV-18 oncogenes (Fig. 6B). Similarly, a slight increase was also observed in the expression levels of the urokinase-type Plasminogen Activator (u-PA) (Fig. 6B). We observed that the expression of heme oxygenase 1 (HO-1/HMOX1), a protein involved in oxidative stress responses, was significantly increased in HaCaT E5/E6/E7 cells compared to levels in the control HaCaT cells (Fig. 6B). More details can be found in Supplementary data (Figure).

## DISCUSSION

Previous studies have demonstrated that the E6 and E7 proteins from HR-HPVs can extend the lifespan of human genital epithelial cells, which function as the host cells of these viruses.^36^ ^,^ ^37^ E6 and E7 proteins play a key role in this process, and they were first identified as disruptors of cell cycle control by affecting the p53 and pRb tumour suppressor pathways, respectively, within host cells.^38^ The simultaneous expression of E6 and E7 is required for cervical cancer development, as these two proteins appear to possess complementary functions.^38^ By contrast, it seems that E5 also acts as an important mediator of oncogenic transformation, as it was observed that the E5 from HPV-16 and -18 is involved in proliferation, migration, invasion, and regulation of the actin cytoskeleton in human cervical cancer cells.^39^ ^,^ ^40^ It has also been observed that the HPV-16 E5 protein protects cells from ultraviolet-B-induced apoptosis and promotes survival of human foreskin keratinocytes by activating PI3K/Akt and MAPK signalling downstream of the EGFR.^41^

In the present study, we used a spontaneously immortalised keratinocyte cell line as a model system to study the effect of HPV oncogenes in the malignant transformation processes. This cell line exhibits traits such as colony formation in soft agar that are reminiscent of transformed cells *in vitro*; however, these cells are non-tumorigenic *in vivo*. Several studies have used this cell line as a model to address the effect of the HPV E6 and E7 oncogenes in different processes associated with cell transformation and immune evasion.^27^ ^,^ ^42^ ^,^ ^43^ Interestingly, HaCaT cells harbour a p53 gene mutational spectrum that is typical of ultraviolet light-induced mutations.^44^ This p53 form is not degraded by the ectopic expression of HPV E6.^45^ Therefore, we can speculate that the effects of HPV 18 oncoproteins on cell signalling, cell proliferation, migration, invasion, and modulation of redox state are regulated, at least in part, by mechanisms that do not rely on p53 degradation. We demonstrated that cooperation among the three HPV-18 oncoproteins (E5/E6/E7) significantly increases HaCaT cell viability and cell proliferation. Our observation is in contrast to the result obtained by Boulenouar and co-workers,^25^ who observed that E5 from HPV-16 impaired the viability of the BeWo choriocarcinoma cell line (known as “trophoblastic-like cells) and cervical cell lines, while E6 and E7 favoured cell growth and neutralised the E5 cytotoxic effect. These differences could be attributed to the different cell lines used or even to the different HPV types analysed. We also observed that HaCaT cells expressing HPV-18 E5/E6/E7 oncogenes exhibited higher invasion potential. This finding is in agreement with previous reports that demonstrated that HPV-16 E5 and E6/E7 enhance trophoblast motility and invasiveness through the induction of epithelial mesenchymal transition (EMT) and associated signalling pathways that function, at least in part, in response to E-cadherin downregulation.^25^ ^,^ ^26^ Furthermore, we detected a higher expression of u-PA and CapG proteins that are involved in invasion and angiogenesis. u-PA is a serine proteinase that has been implicated in the pathogenesis of several epithelial tumours, and it is well known that HPV 16-induced transformation of keratinocytes is associated with upregulation of u-PA expression. In conjunction with other proteinases, u-PA plays an important role in the ability of HPV 16-transformed keratinocytes to penetrate artificial basement membranes.^46^ By n contrast, CapG was previously reported to contribute to tumour invasion and metastasis in multiple human cancers.^47^

Inflammation and oxidative stress are two major cofactors that could also contribute to malignant transformation.^48^ About the pathogenesis of HPV infection, inflammation does not appear to play a central role during the initial stages, as the virus infects the basal cells that are not in direct contact with the circulating immune cells.^49^ However, when persistent infection is established, chronic inflammation could be favoured, and this could, in turn, induce an imbalance between ROS and antioxidant production by the infected cell. In this context, when ROS levels overcome the antioxidant defences of the cell, they induce oxidative stress.^50^ Our observations demonstrated that when expressed together, the three HPV-18 oncogenes induce higher endogenous ROS levels compared to those found in control HaCaT cells and in cells containing E5 and E6/E7 oncogenes. These results are in agreement with previous observations that revealed an acute increase in ROS levels in keratinocytes upon infection with HPV-16.^49^ For example, it has been shown that the E6 small isoform E6* increases oxidative stress and induces DNA damage.^49^ Additionally, another study revealed that HPV-16 E6 and E7 oncoproteins induce oxidative stress and DNA damage in head and neck cancer cells.^48^ Tumour cells are more metabolically active, and this increased activity is associated with the formation of larger amounts of ROS such as hydrogen peroxide.^51^ Antioxidant enzymes such as catalase and peroxiredoxin are critical for maintaining low oxidant levels, and they act as antioxidants in different organelles and at different levels of ROS.^52^ ^,^ ^53^ Our observations revealed a statistically significant increase in catalase activity in HaCaT cells expressing HPV-18 E5 alone compared to the levels in cells expressing HPV-18 E6/E7 and E5/E6/E7 oncogenes. This could partially explain the observation that cells containing E5 of HPV-18 produced significantly lower levels of intracellular ROS. Additionally, it is worth mentioning that in HPV infections, this protein is never expressed alone and is instead likely co-exist with other viral proteins such as E6 and E7. Interestingly, a recent study detailed the presence of higher catalase levels in epithelial organo-typic cultures established from primary keratinocytes expressing HPV16 E6/E7 oncogenes compared to the levels in those that were seeded from keratinocytes transduced with pLXSN empty vector.^54^ However, in this study, the authors did not measure catalase activity. In contrast, Cruz-Gregorio and co-workers demonstrated that expression of HR-HPVs E6/E7 does not alter ROS production or catalase activation in C33A cells.^35^ Taken together, the observations described above reveal the existence of variable effects of the HPV oncogenes on the expression and/or activity of catalase in different cell systems. Further studies are needed to understand the impact of oncogenes from different HPV types on this important cellular protein and to determine the role of catalase in HPV-mediated pathologies.

By decreasing the levels of hydrogen peroxide, antioxidant enzymes prevent the formation of oxidising free radicals, such as hydroxyl radicals, in cells. In our study, we observed an increased ROS production in cells containing the three viral oncogenes that were associated with increases in cytosolic antioxidant defences such as peroxiredoxin and glutathione.^55^ Although this enhanced production of oxidants may be associated with cell proliferation, long-term exposure to higher levels of oxidants will ultimately lead to DNA damage and potential malignant transformations. In contrast to these observations and in agreement with Cruz-Gregorio et al., we observed similar levels of GSH in HaCaT cells transduced with HPV-18 E6/E7 compared to levels present in parental HaCaT cells. We also observed a significant increment in GSH and PRX1 levels in cells expressing E5/E6/E7 compared to the levels observed in cells expressing E5 or E6/E7 or in HaCaT control cells, suggesting that these cells respond to higher amounts of intracellular ROS and an elevated oxidative environment by increasing their antioxidant defences. Consistent with this, we found higher expression levels of HO-1/HMOX1, a protein associated with ROS/RNS-driven oxidative stress responses, in E5, E6, and E7-expressing cells.^56^ This result is in agreement with Cabeça et al., who showed that cultures expressing HPV16 E6 and E7 proteins upregulated the expression of a number of proteins that are related to apoptosis and ROS antioxidant system function such as catalase and HO-1/HMOX1 compared to levels in control cells.^54^

Finally, by proteome profiling we observed that FGF basic, a transcription factor that possesses a ubiquitous role in normal cell growth, survival, differentiation, angiogenesis, and in tumour development,^57^ was overexpressed in HaCaT cells that were transduced with E5, E6, and E7 from HPV-18. By contrast, in E5, E6, and E7-expressing cells, we detected lower levels of Fox01/FKHR, an important transcriptional regulator of cell proliferation. This finding agrees with previous studies that suggest that Fox01/FKHR plays a vital role in inhibiting cervical cancer development by inducing cell-cycle arrest, ultimately suggesting a tumour suppressor function for this protein.^58^

A plausible mechanism that could partially explain our observations may involve the action of HPV-18 E5/E6/E7 oncoproteins in promoting a mild increase in intracellular ROS that could favour cellular proliferation, invasiveness, and an improvement in the antioxidant defences. Several lines of evidence support this. For example, it was observed that the HPV-16 E6 small isoform E6* increases oxidative stress in cervical cancer cell lines and in normal keratinocytes.^48^ Additionally, others authors have demonstrated that HPV-16 E6 and E7 oncoproteins induce oxidative stress and also cause DNA damage in head and neck cancer cells.^47^

Furthermore, ROS appear to activate the *epidermal growth factor* (EGF) and platelet-derived growth factor (*PDGF*) receptors to activate RAS and lead to the subsequent activation of the ERK pathway to promote cell proliferation and differentiation.^59^ ^,^ ^60^ Additionally, it has been reported that ROS also promotes the stabilisation of the Hypoxia-inducible factor protein 1 (HIF-1α) that is involved in neovascularisation and angiogenesis.^61^ ^,^ ^62^ Finally, an increase in ROS levels may induce the activation of Nuclear factor-erythroid 2 p45-related factor 2 (Nrf2), which in turn increases glutathione biosynthesis, PRX1 expression,^63^ and HO-1/HMOX1 expression to function as another anti-oxidant response gene.^64^

Based on our results and the previously published data, we hypothesize that the HPV-18 E5 oncoprotein moderately increases oxidative stress by increasing the intracellular levels of ROS in cell expressing E6 and E7 oncogenes, and through this mechanism, it triggers the activation of different cellular processes such as cell proliferation and invasion. ROS may in turn augment PRX1 expression, HO-1/HMOX1 expression, and GSH levels through Nrf2 activation; however, further functional detailed studies are be necessary to validate this hypothesis and to shed light on this issue.
