# Supplementary material for: Human papillomavirus type 18 E5 oncoprotein cooperates with E6 and E7 in promoting cell viability and invasion and in modulating the cellular redox state
Source: Mem Inst Oswaldo Cruz. 2020 Mar 16;115:e190405. doi: 10.1590/0074-02760190405 (PMC7066992; doi:10.1590/0074-02760190405)
Supplement: Supplementary file 1 [file 1678-8060-mioc-115-e190405-s.pdf]

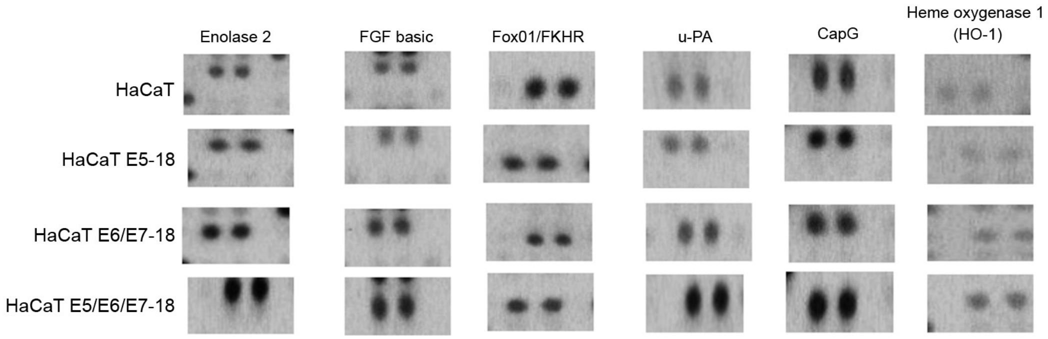

Expression profiles of different proteins involved in signalling pathways related to cancer. Expression levels of different proteins in immortalised HaCaT cells transduced with viral oncogenes E5, E6, and E7 from human papillomavirus (HPV)-18 compared to those of control cells as assessed by the Human XL Oncology Array Kit (R&D Systems, MN, USA, ARY026). All experiments were performed in duplicate.

CC BY
